# The changes in levels of blood cortisol, glucose, and oxygen saturation in type 2 diabetic patients during tooth extraction

**DOI:** 10.1002/cre2.641

**Published:** 2022-07-31

**Authors:** Zana Agani, Jehona Ahmedi, Resmije Ademi Abdyli, Mergime Prekazi Loxha, Vjosa Hamiti‐Krasniqi, Aida Rexhepi, David Stubljar

**Affiliations:** ^1^ Department of Oral Surgery University of Prishtina Prishtina Kosovo; ^2^ Department of Maxillofacial Surgery University of Prishtina Prishtina Kosovo; ^3^ UBT College Higher Education Institution Prishtina Kosovo; ^4^ In‐medico, Research and Development Metlika Slovenia

**Keywords:** cortisol, diabetes, glucose, pain, stress, tooth extraction

## Abstract

**Objectives:**

The extraction of a tooth exacerbates the stress in diabetic patients leading to diabetic complications so the aim was to evaluate the changes in blood cortisol, glucose, and oxygen saturation in type 2 diabetic patients during tooth extraction to pay special attention during a routine surgical procedure.

**Materials and Methods:**

The research included 40 patients with type 2 diabetes with indications of tooth extraction. They were divided into two subgroups by 20 participants and split according to local anesthesia (lidocaine with additional adrenaline or lidocaine only). Cortisol, blood sugar, blood pressure, arterial pulse, and blood oxygen saturation were measured. Patients were also evaluated for their sensitivity to pain through the Visual Analog Scale (VAS).

**Results:**

Cortisol and glucose levels scientifically increased throughout the procedure. Meanwhile, systolic, and diastolic blood pressure and saturation showed no difference between the measurements during and after tooth extraction (*p* = .280; *p* = .090; *p* = .590, respectively). Most patients (60.0%) felt no pain during/after the procedure. None of the subjects was feeling more pain than 30 points by VAS. The comparison between groups receiving lidocaine showed no statistical differences when adding adrenaline to lidocaine. Evaluation of pain by VAS showed that more patients felt pain when they were receiving lidocaine without adrenaline.

**Conclusions:**

Diabetic patients require a more cautious approach when undergoing teeth extractions despite it being a routine procedure.

## 1 INTRODUCTION

Patients who undergo oral surgery produce high levels of stress. In such circumstances, special attention needs to be paid to patients with diabetes mellitus (diabetes), which belongs to the group of metabolic diseases, as it is a condition that might cause complications after routine tooth extraction. Diabetes is a chronic disease with distinguished inflammation‐related processes and is characterized by hyperglycemia. Elevated blood glucose levels have a negative influence on inflammatory responses. Diabetes leads to dry mouth resulting in caries and increases the probabilities of periodontitis, candidiasis, glossodynia, dysgeusia, and temporomandibular joint disorders. For instance, dental plaque can lead to more severe conditions such as gingivitis and periodontitis in diabetic patients (Gazal, 2020; Muriach et al., 2014).

Hyperglycemia is not only part of inflammation processes but can contribute to impaired healing after infection and continues undersurface inflammation with decreased levels of inflammatory biomarkers (Herder & Hermanns, 2019; Prasad et al., 2020). Thus, the extraction of teeth despite being a routine surgical procedure can cause severe after‐surgical reactions for diabetic patients who have been suffering from chronic periodontitis or periapical periodontitis (Lontchi‐Yimagou et al., 2013). The stress reaction during and after the tooth extraction is much larger compared to individuals without interventions or even compared to any other dental procedure (Herder & Hermanns, 2019). The role of several biomarkers released during periodontitis and oral diseases which have been related to risk factors of oral health have been evaluated in the literature. Gingival biotypes either thin or thick have been associated with the type of malocclusion. A higher prevalence of thick gingival biotype was detected in a patient with class II malocclusion and a slightly lower prevalence of thin gingival biotype in a patient with class I malocclusion (Matarese et al., 2016). Genetic polymorphisms also define the evolution or occurrence of oral diseases. For instance, genetic polymorphisms of the methylenetetrahydrofolate reductase (MTHFR) enzyme influence DNA methylation, and alterations of genes contribute to oral squamous cell cancer development. MTHFR C677T and A1298C gene polymorphisms on DNA methylation and site‐specific methylation on p16 and O6‐methylguanine‐DNA methyltransferase gene promoters showed that CT/AC and TT/AA genotypes were significantly higher in cancer patients (Ferlazzo et al., 2017). Moreover, in relation to diabetes, nod‐like receptor family pyrin domain‐containing protein‐3 (NLRP3) complex inflammasome has been identified to contribute to the development of both, periodontitis and diabetes. Patients with periodontitis and patients periodontitis and accompanied by type‐II diabetes had higher serum and salivary NLRP3 concentrations when compared to healthy individuals or patients with diabetes only (Isola et al., 2022).

Therefore, it is important for both dental practitioners to keep in mind the hyperglycemia in diabetic patients and have a better understanding of the factors affecting glycaemic control to improve the management of diabetic patients in the dental office (Cabanillas‐Balsera et al., 2019; Khumaedi et al., 2019). Special attention should be focused on diabetic patients to determine changes in the following biomarkers such as glucose level during tooth extraction, the level of the stress hormone cortisol in serum, arterial blood pressure, and arterial pulse, and to compare the effectiveness of the usage of lidocaine with adrenaline in comparison with lidocaine without adrenaline during the tooth extraction. In the literature, we still see that dental practitioners are not focusing on diabetic disease and are incapable of making a clinical decisions due to their poor knowledge. There is a lack of provided information for diabetic patients, so it is crucial to publish reports in this regard. The extraction of tooth exacerbates the stress in diabetic patients leading to hypo‐ or hyperglycemia thus, the aim of this study was to evaluate the changes in all these parameters (level of blood cortisol, glucose, and oxygen saturation) in type 2 diabetic patients during tooth extraction in order to pay special attention to diabetic patients during a routine surgical procedure.

## 2 METHODS

This study was conducted at the Oral Surgery Clinic of Clinical Stomatological University Center in Pristina, Kosovo. The research was approved by the Ethics Committee of the Faculty of Medicine and performed with the principles of medical ethics according to the Helsinki Declaration on Human Research. The participants gave written consent for their participation in the study. This prospective clinical trial included 40 patients with type 2 diabetes whose blood glucose values did not exceed the value of 10 mmol/L and needed a tooth extraction. Patients with any condition that might impact different immune and physiological responses to tooth extraction were excluded from the analysis. Such patients were allergic reactions to local anesthetics, with hypertension >165/95 mmHg, patients with diabetes with blood glucose values above 10 mmol/L, patients with several teeth extractions, thyrotoxicosis, patients under immunosuppressive therapy, bronchial asthma, type 1 diabetes, tuberculosis, epilepsy, anemia, coagulopathies, hepatitis C, pregnant women, sexually transmitted diseases, liver disease, or any other systemic diseases were not included in the study.

### 2.1 Grouping of patients

Data on patients were collected through interviews taking extensive anamnestic data and providing the classification of patients. The patients were then divided into two subgroups of 20 participants and split according to local anesthesia. The first group received 2 ml of 2% lidocaine with additional 1:100.000 adrenaline, while the second subgroup received 2 ml of 2% lidocaine without adrenaline. The maximum amount of vasoconstrictor was 4 ml, that is, the amount of 0.04 mg vasoconstrictor was not exceeded in one session. If there was a need for a higher dose of anesthetic, then the anesthesia was without the use of a vasoconstrictor.

Patients were measured for serum cortisol levels, blood sugar, blood pressure, arterial pulse, and blood oxygen saturation. Furthermore, patients were also evaluated for their sensitivity to pain through a probe and the Visual Analog Scale (VAS).

### 2.2 Biological stress marker evaluation

The biological stress marker was repeatedly evaluated three times, so several blood samples were taken. The first blood sample was taken 30 min before the application of anesthesia, the second sample was taken during the intervention, and the third sample was taken 30 min after the intervention. Venous blood (3 ml) was withdrawn within EDTA vacuum tube. The collected blood was centrifugated, so the blood separated the serum from the plasma. The samples were stored at a temperature of −18°C until measurements. The level of the stress hormone cortisol was determined by radioimmunoassay method. Blood samples were analyzed in the Endocrinology Laboratory of the Institute of Physiology at the Clinical University Center of Kosovo. Normal values of cortisol were established within the range of 260–720 nM in the morning, and 50–350 nM in the evening.

### 2.3 Measurements of blood sugar, blood pressure, pulse, and oxygen saturation

The measurement of blood sugar was done with blood glucose monitor 3 times. On the day of extraction, the first measurement was performed 30 min before the application of the anesthetic, the second measurement was made during the intervention, and the third measurement was made 30 min after the completion of the tooth extraction. Normal blood glucose values were established as 3–6 mmol/L or 60–130 mg/dl.

The blood pressure was measured three times. The first measurement was made 30 min before the application of the anesthetic, the second measurement was made during the intervention, and the third measurement was made 30 min after the completion of the tooth extraction. The blood pressure measurement was performed using the Spengler manual device by the nurse placing the cuff on the left hand. The bolus was blown to 220 mmHg and then the arterial pressure values were read. Normal blood pressure was established at 120/80 mmHg.

The measurement of the arterial pulse and the saturation of the oxygen in the blood was done with the poly oximeter NONIN Onyx 9500. An arterial pulse measurement and blood oxygen saturation were performed three times. The measurements on the day of extraction were performed 30 min before the application of the anesthetic, the second measurement was made during the intervention, and the third measurement was made 30 min after tooth extraction. The measurements were performed by a nurse by placing the meter on the left hand, so that the forearm and upper arm were at an angle of 90 degrees, relaxed, and on a solid base. Normal arterial pulse rates ranged from 60 to 120 beats per minute, while normal blood oxygen saturation values ranged from 96% to 98%.

### 2.4 Evaluation of pain

The pain was evaluated by the VAS. After the application of the anesthetic before the extraction, a test with a probe was measured objectively whether the patient had sensitivity in the dental region of the tooth that needed extraction. It recorded if the patients were having or did not have sensitivity. The measuring point for the probe was the soft tissue around the tooth and the site of the tooth. After tooth extraction, the patient indicated the sensitivity during the intervention on a scale from 0 to 100, where 0 represented a complete lack of pain, and 100 the presence of maximum pain.

### 2.5 Statistical analysis

Statistical analyses of data were performed using the software package SPSS 21 (IBM). Univariate analysis was used to compare the continuous variables by *t*‐test for the data with normal distribution, or Wilcoxon rank sum test for abnormally distributed data. Statistical differences were significant at *p* < .05.

## 3 RESULTS

Overall, 40 type 2 diabetic patients were enrolled in the analysis that underwent measurements of blood glucose, blood pressure, heart rate, and oxygen saturation at three different time points. The measurements before, during, and after tooth extraction are presented in Table 1.

**Table 1:** Measurements on the day of the extraction

	30 min before anesthesia (*N* = 40)	During tooth extraction (*N* = 40)	30 min after tooth extraction (*N* = 40)	*p* Value
Cortisol (nM)	335.18 ± 112.01	363.7 ± 138.47	385.15 ± 146.53	<.001
Glucose (mmol/L)	7.66 ± 2.76	8.59 ± 3.04	9.32 ± 3.35	<.001
Systolic BP (mmHg)	139.88 ± 18.96	133.13 ± 17.78	131.75 ± 16.78	<.001
Diastolic BP (mmHg)	85.00 ± 9.34	81.50 ± 7.78	79.63 ± 8.65	<.001
Pulse (/min)	82.23 ± 12.01	80.50 ± 13.61	82.13 ± 15.51	.370
SO_2_ (%)	97.0 ± 1.0	96.0 ± 2.0	96.0 ± 2.0	.020

Abbreviation: BP, blood pressure.

There were statistically significant differences in measurement according to three time points for cortisol, glucose, blood pressure, and saturation, but not heart rate. The post hoc analysis of all time points showed that cortisol levels and glucose levels scientifically increased throughout the procedure (data not shown). Meanwhile, systolic, and diastolic blood pressure showed no difference between the measurements during and after tooth extraction (*p* = .280 and *p* = .090, respectively). The same has been observed for saturation in comparison between measurements during and after the procedure (*p* = .590).

The pain was evaluated by VAS ranging from 0 to 100 points. The majority of patients (60.0%) were evaluated and felt no pain (Table 2). None of the subjects included in the study was feeling more pain than equivalent to 30 points by VAS.

**Table 2:** Level of pain during/after the tooth extraction

VAS	Patients (*N* = 40)	%
0	24	60.0
10	3	7.5
20	8	20.0
30	5	12.5

Abbreviation: VAS, Visual Analog Scale.

Twenty patients received local anesthesia of lidocaine with additional adrenaline, and the other 20 patients received only lidocaine. The comparison between the two groups is presented in Table 3. Wilcoxon rank sum test showed no statistical differences when adding adrenaline to lidocaine as local anesthesia. Evaluation of pain by VAS showed that more patients felt pain when they were receiving lidocaine without adrenaline, but the difference in the number of patients was not statistically significant.

**Table 3:** Rank analysis using the Wilcoxon rank sum test for the evaluation of parameters when using lidocaine with adrenaline in comparison with lidocaine without adrenaline during and after the tooth extraction

		Lidocaine with adrenaline (*n* = 20)	Lidocaine without adrenaline (*n* = 20)	*p* Value
After 30 min	Pain (#patients)	6 (30.0%)	10 (50.0%)	.190^a^
During	Cortisol	385.0	435.0	.490^b^
After 30 min	Cortisol	375.5	444.5	.350^b^
During	Glucose	392.5	427.5	.640^b^
After 30 min	Glucose	395.0	425.0	.680^b^
During	Systolic BP	407.5	412.5	.950^b^
After 30 min	Systolic BP	405.5	414.5	.900^b^
During	Diastolic BP	380.0	440.0	.420^b^
After 30 min	Diastolic BP	416.5	403.5	.860^b^
During	Pulse	441.5	378.5	.390^b^
After 30 min	Pulse	420.5	399.5	.780^b^
During	SO_2_	430.5	389.5	.580^b^
After 30 min	SO_2_	437.0	383.0	.470^b^

^a^ Pearson's *χ* ^2^ test.

^b^ Wilcoxon rank sum test.

Analysis of correlations showed that cortisol levels during the procedure were not correlated with the choice of anesthesia (*p* = .830) nor were there associations with the level of pain (*p* = .990), as well as not in the interaction between anesthesia and level of pain (*p* = .630). The same was observed for cortisol levels 30 min after the tooth extraction. No statistical correlation when comparing the types of anesthesia (*p* = .590), nor were there associations with the level of pain (*p* = .960) or in interactions between anesthesia and level of pain (*p* = .580).

## 4 DISCUSSION

Stress can rapidly affect diabetes, exacerbating and worsening the general condition. The psychological response to severe stress causes activation of the adrenal pituitary axis and hypothalamus causing various endocrine abnormalities such as elevated cortisol, and decreased or elevated sensory steroids, which antagonize the action of insulin. This is very important due to the impact of elevated blood glucose levels and the development of complications of diabetes Gazal (2020). Patients who undergo tooth extraction are exposed to stress because of undergoing dental procedures. The aim was to therefore evaluate the changes in level of blood cortisol, glucose, and oxygen saturation in type 2 diabetic patients during tooth extraction.

The release of monogenic catecholamines because of stress and pain during tooth extraction reduces glucose metabolism and increases mobilization and fat metabolism. Decreased glucose metabolism causes an increase in blood glucose levels, and in blood fat levels which affect insulin resistance. The body produces a number of stressful hormones such as cortisol and glucagon, which trigger the release of glucose from the liver; making glucose levels rise significantly (Aggarwal et al., 2019; Saeb et al., 2019). Increased glucose levels of course lead to complications even after a routine procedure, such as tooth extraction. Thus, dental practitioners need to keep in mind hyperglycemia in diabetic patients (Cabanillas‐Balsera et al., 2019). Extraction of teeth must be performed in diabetics within the safe range of blood glucose levels as uncontrolled diabetes triggers osteonecrosis of the jaws (Wang et al., 2018) or dental caries, periodontal disease, and xerostomia (Almusawi et al., 2018).

Our data noticed a significant increase in blood glucose levels during tooth extraction in diabetic patients and coincide with the data of Tily (Tily & Thomas, 2007). The author compared changes in blood glucose levels in patients who have received hypoglycaemic drugs with those who have not and found a firstly significant increase in glucose levels, but also the changes between the groups were significant and much higher in patients who have not received hypoglycaemic therapy.

Although there is evidence that adrenaline has a hyperglycemic effect on blood glucose values, in our study patients who received an addition of adrenaline under anesthesia during and 30 min after tooth extraction, showed no significant difference in values of glucose. Our data match those of Bortoluzzi (Bortoluzzi et al., 2010) who measured blood glucose levels in healthy patients during routine dental treatment by comparing anesthesia with or without adrenaline and found that there were no significant differences between the groups.

People with diabetes experience a reduction in the blood supply to tissues. For instance, poor circulation can cause blood stasis in the periodontal tissues around the teeth. Insufficient blood supply causes periodontal tissue to become starved of oxygen. A low blood oxygen level can cause overstimulation for the osteoclasts and as a consequence, the bone will get absorbed and teeth become mobile (Gazal, 2020). Regarding the changes in SO_2_ values, 30 min before, during, and 30 min after tooth extraction, there was a significant difference in the stated relation, but not during and after the extraction. The average value of SO_2_ during extraction was reduced to the value 30 min before extraction but then stayed the same 30 min after the procedure.

Goetsch et al. (1990), in patients with insulin‐independent diabetes, investigated the effect of acute stress on blood glucose levels and hemodynamic parameters through home 12‐day monitoring. When facing patients with stressful conditions, a significant rise in blood glucose levels, as well as an increase in arterial pulse and blood pressure was noted. Variations in glucose levels in dental treatments have been the subject of research and controversy in the literature. A review by Gazel (2020) determined the maximum acceptable level of blood glucose for tooth extraction in diabetics by establishing fasting blood glucose level at 180 mg/dl (10 mmol/L) for tooth extraction. And blood glucose level of 234 mg/dl (13 mmol/L) was established as a cut‐off for an emergency tooth extraction, thus avoiding hyperglycemia and hypoglycemia.

Meanwhile, Nakamura et al. (2001) observed changes in arterial pressure, plasma catecholamines, glucose, and insulin concentration in 11 normotensive patients who underwent an increase in systolic arterial pressure, arterial pulse, and pulmonary artery disease. The author noted that the concentration of adrenaline increased very quickly after the application of the local anesthetic almost at the same time as he found an increase in blood glucose levels, which means that there is a close relationship between these two variables. This was not observed in our case. Because their study was performed in normotensive patients, this rise in glucose is not significant and is not clinically relevant due to the action of the body's compensatory and regulatory mechanisms. As mentioned, the vascular parameters systolic, diastolic pressure, arterial pulse, and SO_2_ changed in measurements made 30 min before extraction, during extraction, and 30 min after extraction. While systolic and diastolic arterial pressures fall during measurements, that is, they were lower after the tooth extraction compared to pre‐extraction, arterial pulse was higher before and after tooth extraction than during the procedure. When considering also the comparison between patients who received or did not receive adrenaline with anesthetic, we did not find any significant change in vascular parameters. Bortoluzzi (Bortoluzzi et al., 2010) revealed that in his research and explained that adrenaline may have double activity beta1 and beta2. Beta1 stimulates an increase in arterial pressure, while beta2 decreases it. Taking that no dynamic increase in arterial pressure was observed because of beta2 activity. Another explanation might also be that hemodynamic alterations measured in plasma samples are very short due to the half‐life of adrenaline breakdown, which is approximately less than 3 min. However, patients who have been given lidocaine with adrenaline were 0.60 times less likely to have objective pain sensitivity than patients who have been given lidocaine without adrenaline. This, of course, can be explained by a more pronounced anesthetic effect when the anesthetic also has a vasoconstrictor. A study in dogs showed that the addition of epinephrine (precursor of adrenaline) to lidocaine prolonged the duration and increased the intensity of the regional block and at the same time no significant difference was noted in regard to sensory blockade (Choquette et al., 2017). Patients with additional adrenaline did feel less pain according to VAS, but we have found no significance between the group. Similar findings were observed in another study, that confirm the reasoning behind the findings, patients who received lidocaine with epinephrine as vasoconstriction agents, showed prolonged local anesthetic blockage due to a combination of pharmacokinetic and pharmacodynamic effects on local anesthetic actions (Ranganath et al., 2021). This is an important takeaway message as the addition of adrenaline is not creating any harm in diabetic patients, but can even add benefit for the patient.

As seen in this analysis the stressful situation of tooth extraction increases parameters that influence the outcome of dental procedures. When a diabetic patient comes for tooth extraction, special precautions must be made. Dental practitioners need to establish if the patient has control over his blood glucose levels. An early morning appointment minimizes the risk of stress‐induced hypoglycemia (Byakodi et al., 2017; Jia et al., 2019) but the patients must not be hypoglycemic during the extraction procedure (de Bedout et al., 2018; Power et al., 2019). Fasting blood glucose level should be at around 180 mg/dl (10 mmol/L) for a tooth extraction avoiding hyperglycemia and hypoglycemia. Patients need to take food and their medications or insulin injections as normal, and despite they are immunocompromised and requiring treatment for infections, they do not need prophylactic antibiotics. However, in cases of uncontrolled patients, antibiotic prophylaxis is required (Power et al., 2019; Zehani et al., 2017). Dental treatment under local anesthesia should be arranged at least with mealtimes (Gazal, 2020). Dentists encounter many uncontrolled diabetic patients, and it is well‐known that they are not knowledgeable about the conditions of their patients (Alshareef et al., 2019). Therefore, dentists need to be well‐prepared and know how to manage and control patients with diabetes (Power et al., 2019).

Our study had limitations. The sample size of patients included in the study was small, as the analysis was performed in only one department and one institute, so more patients who might be willing to participate were hard to get. The clinical benefit of further studies in this field could give even more knowledge and perhaps someday trigger more tailored treatment strategies for patients with diabetes. Perhaps even a better understanding of subclinical inflammation and pathogenesis may help to identify the risk factors dental practitioners are facing when diabetic patients are coming to a chair.

## 5 CONCLUSION

Stress during tooth extraction elevates glucose levels in the blood and blood pressure of patients with type 2 diabetes. Within this study, we can conclude that diabetic patients require a more cautious approach when undergoing teeth extractions despite being a routine procedure as we want to minimize the impact of their basal disease on the outcomes and potential complications due to hypo‐ or hyperglycemia. Most importantly from a clinical perspective, the addition of adrenaline did not contribute significantly to improving clinical parameters, but clearly showed the trend toward less pain in patients who were receiving lidocaine with adrenaline adding clinical benefit for them.

## AUTHOR CONTRIBUTIONS

*Manuscript preparation, data collection*: Zana Agani and Jehona Ahmedi. *Data collection, data interpretation, literature search*: Resmije Ademi Abdyli. *Study design, final approval of the manuscript*: Mergime Prekazi Loxha. *Data collection, data interpretation, statistical analyses*: Vjosa Hamiti‐Krasniqi and Aida Rexhepi. *Statistical analyses, final approval of the manuscript*: David Stubljar.

## CONFLICT OF INTEREST

The authors declare no conflict of interest.
